# Hereditary tyrosinemia type I–associated mutations in fumarylacetoacetate hydrolase reduce the enzyme stability and increase its aggregation rate

**DOI:** 10.1074/jbc.RA119.009367

**Published:** 2019-07-12

**Authors:** Iratxe Macias, Ana Laín, Ganeko Bernardo-Seisdedos, David Gil, Esperanza Gonzalez, Juan M. Falcon-Perez, Oscar Millet

**Affiliations:** ‡ Protein Stability and Inherited Disease Laboratory, CIC bioGUNE, Bizkaia Technology Park, 48160 Derio, Bizkaia, Spain; § Electron Microscopy Platform, CIC bioGUNE, Bizkaia Technology Park, 48160 Derio, Bizkaia, Spain; ¶ Exosomes Laboratory, CIC bioGUNE, Bizkaia Technology Park, 48160 Derio, Bizkaia, Spain; ‖ IKERBASQUE, Basque Foundation for Science, Bilbao, 48013 Spain

**Keywords:** protein aggregation, protein stability, enzyme mutation, nuclear magnetic resonance (NMR), biophysics, tyrosine, fumaryl acetoacetate hydrolase, rare disease, tyrosinemia type I

## Abstract

More than 100 mutations in the gene encoding fumarylacetoacetate hydrolase (FAH) cause hereditary tyrosinemia type I (HT1), a metabolic disorder characterized by elevated blood levels of tyrosine. Some of these mutations are known to decrease FAH catalytic activity, but the mechanisms of FAH mutation–induced pathogenicity remain poorly understood. Here, using diffusion ordered NMR spectroscopy, cryo-EM, and CD analyses, along with site-directed mutagenesis, enzymatic assays, and molecular dynamics simulations, we investigated the putative role of thermodynamic and kinetic stability in WT FAH and a representative set of 19 missense mutations identified in individuals with HT1. We found that at physiological temperatures and concentrations, WT FAH is in equilibrium between a catalytically active dimer and a monomeric species, with the latter being inactive and prone to oligomerization and aggregation. We also found that the majority of the deleterious mutations reduce the kinetic stability of the enzyme and always accelerate the FAH aggregation pathway. Depending mainly on the position of the amino acid in the structure, pathogenic mutations either reduced the dimer population or decreased the energy barrier that separates the monomer from the aggregate. The mechanistic insights reported here pave the way for the development of pharmacological chaperones that target FAH to tackle the severe disease HT1.

## Introduction

Hereditary tyrosinemia type I (HT1, OMIM 276700)^3^ is an autosomal recessive rare disorder caused by a deficiency in fumarylacetoacetate hydrolase (FAH, EC 3.7.1.2), the last enzyme in the tyrosine catabolism pathway (1). HT1 is found worldwide except in Central America and Oceania (2), and it shows a relatively low incidence, with one affected individual in ∼100.000 healthy people, on average (3). The disease is characterized by progressive liver disease and renal tubular dysfunction that leads to hypophosphatemic rickets (4). It mainly targets hepatocytes and renal proximal tubular epithelium (5), where the enzyme is predominantly expressed.

At the molecular level, a reduced activity of FAH upon mutation leads to the accumulation of upstream metabolites like fumarylacetoacetate (FAA) and maleylacetoacetate, which are subsequently converted to succinylacetoacetate, then decarboxylated to succinylacetone, and finally accumulated in many body tissues. These metabolites are highly reactive with electrophilic compounds and ultimately responsible for the progressive hepatic, renal, and neurological damages (6).

FAH is a cytosolic homodimer with two 46-kDa subunits conformed by a 120-residue N-terminal domain (N-term) and a 300-residue C-terminal domain (C-term). The N-term has an SH3-like fold and is supposed to play a regulatory role (7, 8), whereas the C-term is defined by a new β-sandwich roll structure that is implicated in metal–ion binding and catalysis, participating in intermolecular interactions at the dimer interface (9). Structural inspection of the complex with the reaction products (10) or inhibitors (8) strongly suggests that the dimer is the sole catalytically active species. In members of the FAH family, a dimer is always in equilibrium with its monomeric form (*K_D_* between 0.1 and 1.6 μm) (10, 11), but FAH oligomerization thermodynamics and its putative functional role have not been studied in detail.

To date, ∼100 mutations have been reported to cause HT1: 45 missense mutations, 23 splice defects, 13 nonsense mutations, 10 deletions, and 4 frameshift alterations (12). C-term concentrates the pathogenic defects (69 *versus* 9 defects), suggesting that deleterious mutations in this domain target functional residues. However, missense mutations may affect the catalytic activity, the equilibrium between the monomer and the dimer, or the protein stability *in vitro* and/or its cellular homeostasis, and the exact mechanism by which a mutation confers pathogenicity to FAH is largely unknown.

Here, we have thoroughly studied the monomer–dimer equilibrium of WT FAH by NMR spectroscopy and CD to show that dimer FAH is the functionally pertinent species, whereas monomer FAH tends to aggregate at physiological conditions. We have also selected and biophysically and biochemically investigated 19 missense mutations found in HT1 patients (Table S1). Mutations are widespread across the structure (Fig. S1), and they become a representative set of the overall pool of FAH mutants, in terms of location, mutation type, relative catalytic activity, and the acquired phenotype in hereditary HT1. The energy landscape obtained for WT-FAH and the mutant set identified two clearly distinct molecular mechanisms for pathogenicity: mutations located at the C-term shift the equilibrium toward the monomer, ultimately triggering aggregation, whereas mutations located in the N-term are likely to produce a significant conformational change that decreases the stability of monomeric FAH.

## Results

### Only the dimeric form of WT FAH is catalytically active

**Figure 1.**
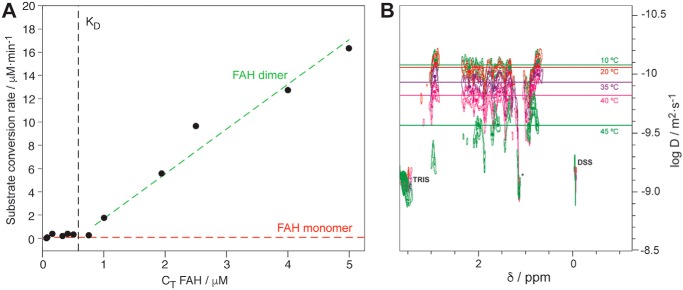
*A*, substrate conversion as a function of the total concentration of FAH (*C*_T_). The *black dashed line* represents the reported *K_D_* value that is consistent with the data. *Red* and *green dashed lines* fit the subset of data in which the majoritarian species correspond to the monomer or the dimer, respectively. *B*, overlap of DOSY spectra for WT FAH at different temperatures, as indicated. Spectra have been normalized to Tris and 4,4-dimethyl-4-silapentane-1-sulfonic acid (*DSS*) because both molecules show no structural variation in this temperature range. The *lines* reflect the diffusion coefficient (log D, *y* axis), whereas the *circles* indicate the positions of the peaks.

First, we have addressed the role of protein's dimerization in the enzymatic activity of WT FAH. For the FAH superfamily, dimeric FAH is the dominant species with a dissociation constant between 1.7 μm (11) and less than 100 nm (10). Analysis of the high-resolution structures strongly suggests that the dimerization is essential for the formation of the catalytic site and optimal for substrate accommodation (10). Heterologous expression and purification of WT human FAH yielded a functional form of the enzyme, capable of converting FAA into fumarate and acetoacetate when following a previously established protocol (13). Fig. 1*A* shows the rate of substrate conversion at varying concentrations of FAH total protein (*C*_T_, 0.1–10 μm) and at a given amount of FAA (2 mm). Clearly, enzymatic conversion is appreciated only for FAH concentrations of 0.5 μm or above, where the dimer population dominates. Accordingly, the NMR measurement of the diffusion coefficient by diffusion ordered spectroscopy (DOSY; Fig. 1*B*) at 20 °C is consistent with a theoretical hydrodynamic diameter of 94 Å (using a Stokes–Einstein diffusion model), in agreement with a dimer species for WT FAH (diameter of the dimer is 82 Å). Interestingly, even at the large concentrations required for the DOSY experiment ([FAH] = 27 μm), the dimer/monomer equilibrium is very sensitive to temperature for WT FAH and, at 37 °C, a significant population of monomer is also detected (Fig. 1*B*).

A second set of reaction rates, measured at multiple substrate concentrations and using 5 μm of FAH at 22 °C, was fit to a Michaelis–Menten model to determine the kinetic parameters for the enzyme (Fig. S2; *K_m_* = 25.2 ± 3 μm and *k*_cat_ = 0.10 ± 0.02 s^−1^). In summary, the experimental data set is consistent with a canonical Michaelis–Menten enzymology for a sole active species (the dimer), in agreement with previous structural analyses and with a *K_D_* of ∼0.5 μm for our protein preparation.

### Monomeric (inactive) WT FAH is unstable and prone to aggregate

**Figure 2.**
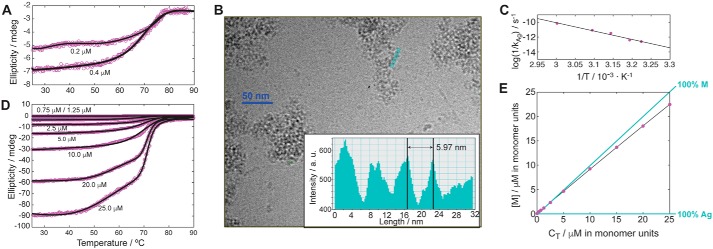
*A* and *D*, CD data for WT FAH at concentrations where the monomer (*A*) or the dimer (*D*) species are majoritarian. *Purple open circles* correspond to the experimental data, whereas *solid black lines* are the best collective fitting to the bimodal thermal denaturation. *B*, representative micrograph from aggregates of WT FAH. The *inset* reflects the average length of the aggregating particles from the *green*-highlighted stretch of particles. *C*, Arrhenius plot for WT FAH aggregation constant (*k*_Ag_) *versus* temperature. The *solid line* corresponds to the best fit of the magnitudes shown in the axes. *E*, plot of the monomer concentration *versus* the total concentration (*C*_T_) of WT FAH. The *lines* where the FAH concentration is monomeric (*M*) or aggregated (*Ag*) are shown in *green*.

Thermal denaturation monitored by CD of freshly obtained FAH at the concentrations of the monomeric form (*C*_T_ < 1 μm) fits well to a single unfolding event and is characterized by a single melting temperature (*T*_*m*_^*C*_T_→0^ = 68.3 ± 0.6 °C) (Fig. 2*A*). However, irreversible refolding (signal recovery of <5%) followed by aggregation was observed upon cooling the sample. Chemical denaturation using guanidinium chloride at a physiological temperature (*T*_0_ = 37 °C) is reversible and provides a free energy of unfolding (Δ*G*_*FU*_^*T*_0_^) of 9.5 ± 0.4 kcal·mol^−1^ (Fig. S3).

The irreversible unfolding may be due to the aggregation of the monomer-like or dimer-like particles in the folded state, the monomer in the unfolded state, or a combination of them. Heating the sample to 80 °C does not increase the amount of precipitate and does not change the remaining ellipticity, strongly suggesting that the unfolded state is not involved in the aggregation process. Furthermore, EM analysis of the aggregates (Fig. 2*B*) shows amorphous oligomerization that results from the aggregation of globular proteins, without any apparent bidimensional order. Micrograph analysis reveals a particle's average size of 60 ± 5 Å, which agrees well with the *monomeric folded* form of FAH (62 Å of maximum diameter).

Considering its instability, we next investigated the time evolution of monomer FAH toward the aggregate (*k*_*Ag*_^*T*_0_,*C*_0_^[*t*]) monitored by CD at physiological temperature and at *C*_0_ = 0.1 μm. The population of monomer decreases exponentially over time because of aggregate formation (Fig. S4 and Table S1). The obtained value is large (*k*_*Ag*_^*T*_0_,*C*_0_^[*t*] = 40). The temperature dependence of the kinetic destabilization of folded FAH at this concentration agrees well with an Arrhenius model (Fig. 2*C*), providing an enthalpic component to the activation energy (*E*_*a*_^*C*_0_^) of 20.9 ± 1.6 kcal/mol. *E*_*a*_^*C*_0_^ is the energy barrier that separates the monomer species from the (more stable) final aggregate. The value found for FAH is close to the ones reported for other proteins like human pancreatic α-amylase (14.4 kcal/mol) (14) or the I27 domain of human cardiac titin (22 kcal/mol) (15) but lower than other enzymes like uroporphyrinogen III synthase (101.5 kcal/mol) (16).

### Dimeric (active) WT FAH is a kinetically stabilized protein

Equivalent thermal denaturation of WT FAH in the concentration range of the dimer (2.5–25 μm) (Fig. 2*D*) does not undergo reversible refolding (signal recovery < 15%) and also results in the formation of macroscopic protein aggregates that precipitate. The denaturation curves are FAH concentration–dependent and bimodal with a first apparent denaturation event characterized by a *T*_m1_ at ∼48 °C and a second thermal melt with a *T*_m2_ of ∼70 °C, largely coincident with *T*_*m*_^*C*_T_→0^. Actually, the concentration dependence affects mainly the first apparent melting event, which becomes more pronounced at increasing concentrations and is proportional to the amount of precipitated protein (Fig. 2*D*). At temperatures at approximately *T*_m1_, the dimer dissociates into the monomer (Fig. 1*B*), consistent with the irreversible aggregation observed.

Altogether, the empirical observations agree well with a model were the *active* dimer (D) is in equilibrium with the *inactive* monomer species (M) which, in turn, evolves either to the unfolding state (U) or irreversibly to a final aggregated state (Ag),

**Scheme 1:**
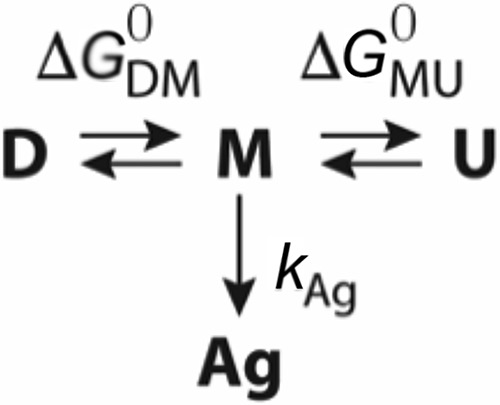

where *G*_*DM*_^0^ and *G*_*MU*_^0^ correspond to the dimer's dissociation and protein's unfolding free energies, respectively, and *k*_Ag_ is the irreversible kinetic rate of aggregation.

Aggregation is a complex process that depends on protein concentration (*C*_T_), time (*t*), and temperature (*T*). The entire data set (Fig. 2, *A* and *D*) can be adjusted to a bimodal version of the linear extrapolation model (17), using a single value for *T*_m2_ and where the first apparent denaturation curve is proportional to the molar fraction of the formed oligomer (χ_A_).

(Eq. 1) $$f\left(\theta ,T\right)=\chi_{A}\cdot f_{1}\left(\theta ,T_{\text{m}1}\right)+f_{2}\left(\theta ,T_{\text{m}2}\right)$$

The modeled curves (*black lines* in Fig. 2, *A* and *D*) show excellent agreement with the experimental data. The magnitude 1 − χ_A_, obtained from the previous fitting, shows a phenomenological exponential dependence with *C*_T_, and it can be used to estimate the phase diagram for aggregation (at *T* ≈ *T*_m1_ and *t* → 0), based on the exponential fitting of the monomer concentration ([M] = (1 − χ_A_)·*C*_T_) *versus* the total concentration of protein *C*_T_. The *diagonal line* in Fig. 2*E* reflects a hypothetical monomeric stable species, whereas WT FAH deviates from the diagonal (*x* = *y*), underlying its tendency to aggregate as a function of *C*_T_.

### HT1 mutants lead to a reduction of the catalytic activity of FAH

To investigate the molecular basis of HT1, we have selected 19 missense mutations reported as pathogenic for this disease in the Human Gene Mutation Database (18) (Table S1). HT1 associated FAH mutations lead to widespread changes across the protein structure (Figs. S1 and S6), and 19 such missense mutations become a representative set of the overall pool of reported HT1-causing FAH mutants, in terms of mutation type, location, and acquired phenotype in HT1 (19).

**Figure 3.**
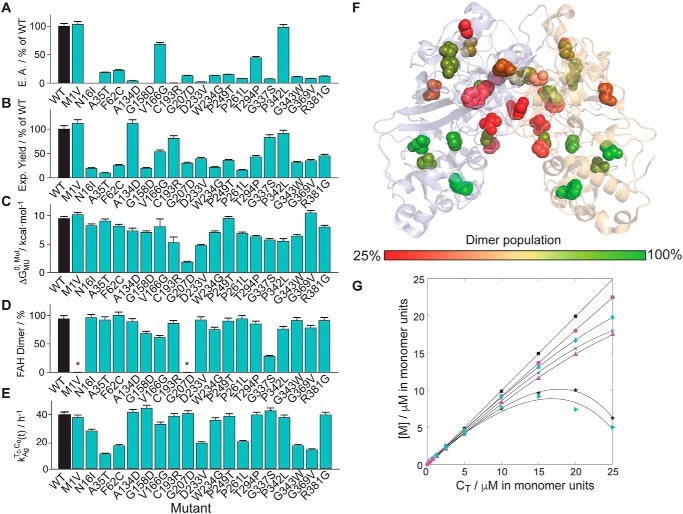
**Biochemical and biophysical properties of the investigated mutants.** *A*, enzyme activity (*E.A.*). *B*, expression (*Exp.*) yield. *C*, unfolding free energy. *D*, dimer content. *E*, aggregation rate of the monomeric species. All the properties are relative values from WT FAH unless otherwise indicated. WT/mutant values are indicated from *black*/*green bars*, respectively. The *error bars* were obtained from duplicate data and error propagation. *F*, fraction of dimer (as compared with WT FAH) plotted in the dimer structure of FAH. The color code is indicated in the *legend. G*, plot of the monomer concentration *versus* the total concentration (*C*_T_) of representative mutants: F62C (*black squares*), WT (*purple circles*), N16I (*green diamonds*), G158D (*crosses*), W234G (*purple triangles*), V166G (*black stars*), and G337S (*green triangles*). The *asterisk* indicates that the data are not available.

First, we evaluated to which extent the pathogenic missense mutations alter the intrinsic catalytic activity of FAH, as compared with WT FAH. Enzymatic assays for the mutated versions of FAH (5 μm) were performed by monitoring the conversion of FAA (250 μm) at 37 °C, compared with the equivalent reaction with WT FAH (Fig. 3*A*). Unsurprisingly, the vast majority of the pathogenic mutants under consideration lead to a depletion of the enzymatic activity, in many cases below 20%. The only exceptions are M1V and P342L, which show a catalytic activity comparable with WT FAH. M1V FAH does not destabilize the protein relative to WT FAH (see below), but a transcription error results in impaired eukaryotic expression (20).

### HT1 mutations retain the thermodynamic stability upon unfolding

Next, we investigated whether the mutation set retains or alters FAH thermodynamic unfolding stability. To do so, WT FAH and the mutant set were overexpressed in *Escherichia coli* for protein production. Yields from preparations, expressed and purified under identical conditions, offer a first phenomenological reporter for the relative protein stability (Fig. 3*B*). In all cases but M1V and A134D, the yield becomes reduced as compared with WT FAH. Indeed, A134D is known to be affected by the allocation of the substrate in the active site (21, 22), causing protein malfunctioning but not necessarily homeostatic problems.

The free energies of unfolding (Δ*G*_*MU*_^0,Mut^) have been obtained from chemical denaturation experiments using urea or guanidinium chloride (Table S2). Alternatively, the *T*_*m*_^*C*_T_→0^ values for the entire set of altered proteins, obtained from thermal melts, have also been used to estimate the stability of their folded monomeric species according to the following equation (23),

(Eq. 2) $$\Delta \Delta G_{\text{MU}}^{0,\text{WT}-\text{Mut}}\approx \Delta H_{\text{MU}}^{0,T_{\text{m}}}\cdot \frac{\Delta T_{\text{m}}}{T_{\text{m}}^{\text{WT}}}$$

where Δ*H*_*MU*_^0,*T*m^ = 75 kcal·mol^−1^ is the enthalpic component at the melting temperature, estimated for WT FAH from the thermal denaturation curve fitting. The good agreement between the two independent experiments validates the use of a single value for the enthalpy at *T*_m_.

The values of (Δ*G*_*MU*_^0,Mut^ are generally lower than WT (Table S1 and Fig. 3*C*). However, all but one show large stabilities upon unfolding, with folded species dominating at physiological temperature (population ≫ 99%). In the case of G207D, the stability is significantly lowered (down to 1.2 kcal·mol^−1^), with a partially populated unfolded state (∼15%).

### Distinct destabilization mechanisms for the mutations in the C-term and in the N-term

Thermal denaturation CD spectra of FAH mutants present very notorious bimodal profiles (Fig. S5), indicating that HT1-causing mutations may be accompanied by FAH dimer disruption, as compared with WT FAH. The population of the dimer species in the mutant-derived proteins was estimated using DOSY spectroscopy (Fig. 3, *D* and *F*). Mutations affecting at the dimer interface (Gr1 group: A134D, G158D, V166G, C193R, W234G, P249T, T294P, G337S, P342L, G369V, and R381G) moderately or significantly shift the equilibrium toward the monomer (Fig. 3*F*), reducing the population of the active dimeric species in solution. One exception is R381G, which does not significantly alter the dimer population. Arg-381 is located at the C-term but relatively far from the dimerization interface. Any case, this result highlights the multifactorial nature of the deleterious mutations.

The phase diagram for this subset of mutants, obtained from the analysis of the thermal denaturation data, shows clear deviations of the monomer (diagonal) line (Fig. 3*G*). MD simulations reveal that these mutations loosen contacts between residues located in the dimerization interface and/or in the binding pocket (*i.e.* Thr-120, Tyr-124, Tyr-128, Arg-162, Asp-344, and others), ultimately debilitating the dimer stability (Fig. S6). Finally, the *E*_a_ for these mutants, measured at concentration where the dimer is populated (*i.e.* 10 μm), decreases as compared with WT FAH (Fig. S3), suggesting that the energy barrier between the dimer and the monomer is affected upon mutation. These changes are likely to be produced only locally at the dimer interface because the CD spectra for these mutants largely coincide with the WT FAH ones (Fig. S7).

Mutations producing changes at the N-term (N16I, A35T, and F62C) and at the C-term, which are distant from the dimerization site but face the C-term (D233V, P261L, and G343W), constitute another set (Gr2 group) and share a different destabilization mechanism. Unlike the Gr1 group, they have dimer populations comparable with WT FAH (Fig. 3, *D* and *F*) and aggregation curves close to the monomeric (diagonal) line (Fig. 3*G*), consistent with their long distance to the dimerization site. However, the *k*_*Ag*_^*T*_0_,*C*_0_^[*t*] measured at physiological temperature and at *C*_0_ = 0.1 μm (Fig. 3*E*) indicate that the monomers from these set of FAH mutants have higher tendency than WT FAH to evolve toward the aggregate species (Fig. S3). In addition, the CD spectra differ from the WT ones (Fig. S7), suggesting a conformational change. According to the CD spectrum, the helical content would be reduced, consistent with a partial disruption of the α-helix of the SH3 domain.

### Interplay between in vitro stability and intracellular homeostasis

To investigate the proteostasis in the cellular environment, overexpressing constructs for FAH (WT and several selected mutants, pCMV6-AC-GFP) were transiently transfected into M1 human fibroblast cell lines. Mutations were selected according to location, stability, and expression yield of the corresponding protein version: A35T and T294P (Gr2 group) are located in the N-term and in the interface between the C-term and the N-term, respectively. According to the *in vitro* results, they should result in a conformational change and increased aggregation. V166G and W234G (Gr1 group) affect the dimerization process, causing the disruption of the active site. The latter one is also related to calcium binding (24), necessary for the catalytic activity.

**Figure 4.**
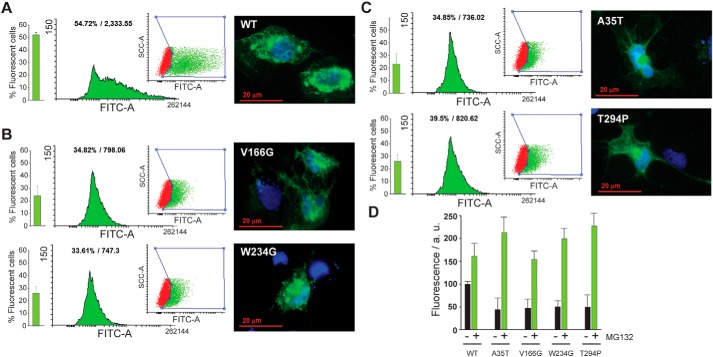
*A–C*, human fibroblastoid M1 cells 24 h after FAH-GFP transfection as measured by FACS. For each FAH variant, the *left bar plot* shows the percentage of fluorescent cells from two independent experiments, whereas the *histograms* show one representative data set. FITC-A shows GFP fluorescence emission; the percentage and median fluorescence intensity of GFP-positive cells are indicated in each condition. Fluorescence microscopy images show representative examples of the protein distribution in the cellular environment. The images show the overlap of the 4′,6′-diamino-2-phenylindole channel (*blue*) with the GFP-sensitive excitation frequency (*green*). Transfection with WT FAH-GFP construct (*A*), Gr1 group mutants (V166G and W234G, *B*), and Gr2 group mutants (A35T and T294P, *C*) is shown. *D*, increase in fluorescence for the M1 FAH-GFP clones after treatment with MG-132. The increase in the fluorescence is due to protein accumulation in the cell caused by proteasome degradation pathway inhibition and is related to the WT FAH basal fluorescence in the absence of proteasomal inhibitor.

Flow cytometry revealed that cells expressing WT FAH show a strong GFP fluorescence emission (Fig. 4*A*). However, intracellular protein levels of cells expressing mutations of FAH decays drastically (Fig. 4, *B* and *C*), indicating that either the expression levels are reduced or that FAH becomes unstable upon mutation and triggers a degradation mechanism in the cell, which is consistent with the thermodynamic data and the reduced expression yield in *E. coli*. In all cases, fluorescence microscopy reveals that the protein is likely located in the cytosol in the form of aggresome. These cytosolic aggregates suggest that the expressed protein has exceeded the protein quality control capacity or solubility limit.

FAH protein levels can be restored upon cell treatment with the proteasome inhibitor MG-132 (Fig. 4*D*), indicating that the natural degradation of the protein occurs through the proteasomal degradation pathway. In all cases, the levels intracellular rescue of protein for the mutants were much higher than for WT FAH, highlighting the exacerbated homeostatic problems introduced with the mutation.

## Discussion

In this paper, we have investigated the role of fumarylacetoacetate hydrolase stability in function and disease. A thorough biophysical and biochemical characterization of WT FAH provides experimental evidence to corroborate the hypothesis that the dimer is the only active species, whereas the monomer remains inactive. This result is totally consistent with the quaternary high-resolution structure, the active site location, and the metallic cofactor binding (10). Intriguingly, only the C-term intervenes in catalysis, and it has been hypothesized that the N-term plays a regulatory role (19). From the energetic point of view, the scenario becomes more complex because FAH is a kinetically stabilized enzyme that undergoes an equilibrium between the functional dimer and an unstable monomeric species, the latter spontaneously evolving toward an aggregate form. Analysis of the EM micrographs suggests that the folded monomer is the main component of the amorphous aggregates.

**Figure 5.**
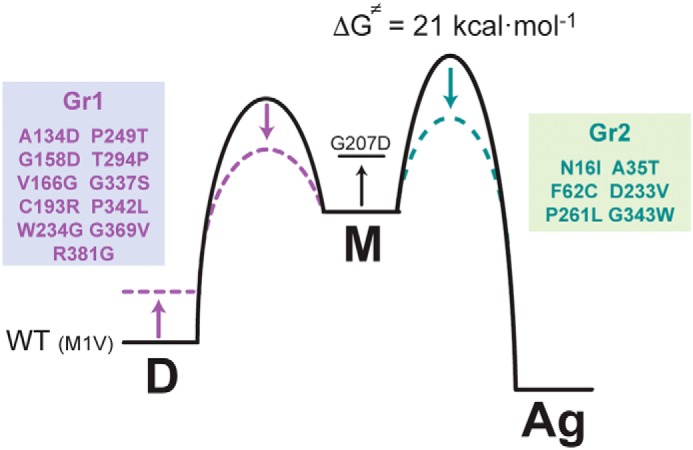
**Energy diagram for WT and M1V FAH (*solid line*), the mutants of the Gr1 group (*purple dashed line*) and the mutants of the Gr2 group (*dashed green line*).** *D*, dimer; *M*, monomer (folded); *Ag*, aggregate. The mutant G207D destabilizes the monomeric form, as indicated. The free energy for the transition state toward aggregation has been calculated from the experimental energy barrier by means of the Eyring equation, whereas the other changes in energy levels are only qualitative estimations, are mutant-dependent, and are not drawn to scale.

The energy landscape for the inactivation–aggregation pathway is schematically represented in Fig. 5, where the experimental activation energy obtained from the Arrhenius equation (Fig. 2*C*) is interpreted in terms of the transition state theory as the free energy of the transition state. The good agreement between the fitted pre-exponential factor and the calculated prefactor when using the Eyring equation (ln*A*_expt_ = 26 s^−1^ *versus* ln*A*_calc_ = 28 s^−1^) supports this assumption. The large value obtained for the energy barrier (21 kcal·mol^−1^) assures that the dimeric species for WT FAH is populated long enough to exert its function.

Some missense mutations in the gene encoding for FAH result in HT1, characterized by elevated blood levels of tyrosine. From the therapeutic point of view, dietary restriction in phenylalanine and tyrosine (25), liver transplantation, and, more recently, an effective inhibitor of 4-hydroxyphenylpyruvic acid dioxygenase (nitisinone) (26) constitute the therapies available, which are rather effective but with uneven success and significant secondary effects and long-term complications for the patients. Thus, this disease requires alternative therapeutic intervention lines that would imply less toxic side effects. To that end, a proper characterization of the pathogenic mechanism induced by the mutation is required, and we have studied a pool of 19 mutations that adequately describe the entire set of HT1-causing missense mutations. All the investigated HT1-producing versions of FAH but G207V report very high stability upon unfolding, indicating that monomeric FAH remains folded and is largely resistant to mutation, in agreement with aggregates being constituted by *folded* monomers only.

Remarkably, despite additional deleterious effects that the mutation may introduce (*i.e.* alterations in substrate binding, catalysis, etc.), all the mutants in the set but M1V (which alters transcription) impair stability through two well-differentiated mechanisms (Fig. 5). Mutants affecting the interface (Gr1 group) primarily destabilize the dimeric species, overpopulating the monomer, which is inactive and unstable, accelerating aggregation. This mechanism alone is able to explain the pathogenic character of the entire Gr1 group, but it is important to emphasize that amino acids from the dimer interface also conform the active site, and it is more than likely that some mutations may also impair substrate binding and/or the catalytic reaction, as previously reported (19).

Mutants producing changes at the N-term or in the C-term interfacing with the N-term (Gr2 group) do not alter the monomer/dimer equilibrium. Instead, they prove to reduce the kinetic stability of the monomer by lowering the energy barrier that separates the monomer from the aggregate. Moreover, CD and NMR experiments suggest a conformational change, consistent with previously reported observations (21). We hypothesize that aggregation occurs through oligomerization of the N-term, perhaps requiring partial unfolding of this moiety.

All the mechanistic information obtained *in vitro* has been corroborated with the experiments in eukaryotic cells, where impaired homeostasis observed upon mutation highlights the putative role of deficient FAH proteostasis for the onset and development of HT1. Such interplay is of a general nature, and it has also been reported for other systems (27–29) as well as for some mutants in FAH (21). Here, the two mechanisms found for the HT1-causing mutations offer two novel putative therapeutic strategies: competitive inhibitors acting as pharmacological chaperones by stabilizing the dimer species should be effective against mutations of the Gr1 group, whereas chemical entities may be able to abrogate FAH oligomerization by binding at the N-term. This second strategy would be especially suitable for mutations enclosed in the Gr2 group.

## Experimental procedures

### Site-directed mutagenesis

Human FAH cDNA (FLJ94596) sequence, registered in the NCBI repository with accession number AK313951.1, was chosen as template. The multiple mutants used in this work were obtained by site-directed mutagenesis performed with the commercial QuikChange Lightning site-directed mutagenesis kit (Agilent Technologies) with custom-made oligonucleotides (Table S3) as PCR primers (Invitrogen).

### Protein production and purification

Protein production was performed using standard protocols previously described (30), with the exception that FAH and GSH transferase ζ1 (GSTZ1) were grown at 37 °C for 16 h, whereas homogentisic acid dioxygenase (HGD) was grown at 25 °C until an *A*_600_ of 0.6 to prevent the formation of inclusion bodies. Protein purification was obtained by nickel–nitrilotriacetic acid resin (Invitrogen) followed by size exclusion chromatography (Hi-Load 26/60 Superdex 75; GE Healthcare) and eluted in 20 mm Tris, 300 mm NaCl, pH 8, for FAH; 20 mm Tris, 500 mm NaCl, pH 7.0, for HGD; and 5 mm HEPES, pH 7.0, with 5% (v/v) glycerol for GSTZ1. Protein quantification was performed by spectrophotometrical measurement (*E*_FAH_(280 nm) = 55,550 m^−1^ cm^−1^; *E*_HGD_(280 nm) = 69,280 m^−1^ cm^−1^; and *E*_GSTZ1_(280 nm) = 19,300 m^−1^ cm^−1^).

### FAA synthesis and purification

The synthesis of fumarylacetoacetate was based on the method of Fernández-Cañón and Peñalva (13). First, HGD was activated for 30 min with 20 mm KHPO_4_, pH 7.4, 2 mm ascorbic acid, and 1 mm FeSO_4_ at room temperature. The solution was then mixed with 20 mm KHPO_4_, pH 7.4, 2 mm ascorbic acid, 0.2 mm FeSO_4_, and 2 mm homogentisic acid. The reaction was incubated at 37 °C for 3 h to achieve full conversion of homogentisic acid into 4-maleylacetoacetic acid. GSTZ1 was then incubated with 20 mm KHPO_4_, pH 7.4, 2 mm ascorbic acid, and 1 mm reduced GSH (Sigma–Aldrich) at room temperature. Once the HGD reaction is finished, 10% (v/v) of volume reaction of 10% (w/v) metaphosphoric acid was poured on ice and left for 10 min to stop the reaction and precipitate the enzymes. The solution was then centrifuged for 10 min at 14,000 rpm, and the supernatant was neutralized with 4 m KOH. Activated GSTZ1 was poured directly to the solution in a proportion of 1:10, and the mixture was left for 2 h at 37 °C, stopped by adding 10% (v/v) of the total volume of 10% (w/v) metaphosphoric acid, and left on ice for 10 min.

FAA purification was performed in a Shimadzu HPLC equipped with SIL20AC HT multisampler, CTO-10AS VP, LC-20AD, CBM-20A, and SPD-M20A modules. The fumarylacetoacetate solution was acidified with acetic acid at pH 3 and loaded into a BDS Hypersil C18 column (250 × 3 mm Teknokroma; Thermo Scientific) in an acetic acid–water (A) and acetonitrile (B) gradient. The gradient program was set at 1 ml/min as follows: 0–10 min; from 0% (v/v) to 20% (v/v) B; 10–10.1 min; from 20% (v/v) to 0% (v/v) B; and remained for 10 min at 0% (v/v) B to re-equilibrate the column before the next injection. Under these conditions the FAA peak elutes at 10 min. The fumarylacetoacetate concentration was quantified using the molar extinction coefficient of 13,500 m^−1^ cm^−1^ at 330 nm.

### Enzymatic assay

The enzymatic activity of FAH is measured by the disappearance of FAA monitored at 330 nm in a Jasco V630 BIO spectrophotometer. The reaction rate, ν, was obtained from the slope of the FAA linear decay. The representation of reaction rate *versus* the substrate concentration was adjusted to a Michaelis–Menten model.

### CD experiments

Purified FAH protein was loaded in a Quartz SUPRASIL cuvette 2 mm thick, from Hellma Analytics. The measurements were done in a JASCO J-810 CD spectrophotometer connected to a JASCO Peltier. For thermodynamic studies, the apparent melting temperature was monitored at 222 nm using temperature scans from 25 to 90 °C to ensure the proper determination of the baselines in both the folded and unfolded states. Temperature increasing rate was 1 °C/min. The data were analyzed using in-house built scripts programmed in Matlab (Simulink) assuming the linear extrapolation method (31): the molar ellipticity at each point of the transition can be described as a linear combination of the expected values for the folded (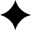
_F_) and unfolded (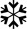
_U_) states. The values for 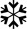
_F_ and 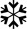
_U_ are obtained from extrapolations of the linear baselines. From these data, we obtained the melting temperature (*T*_m_) and ΔH° of WT and mutant proteins. Chemical denaturation using guanidinium chloride or urea (0–7 m) was measured in steps of 0.1 m cosolute and analyzed using an equivalent fitting strategy. For kinetic stability studies, the decay in ellipticity signal at 222 nm was monitored at a constant temperature over the time. The isothermal aggregation rate (*k*_Ag_) was determined for each of the FAH mutant and WT proteins at different temperatures.

### NMR experiments

All NMR experiments were performed on a Bruker Avance III 800 MHz NMR spectrometer equipped with triple resonance ^1^H,^13^C,^15^N-cryoprobe. All experiments were performed at 298 and 318 K. Chemical shifts are given in ppm with respect to 4,4-dimethyl-4-silapentane-1-sulfonic acid as internal reference. The 2D-DOSY experiments were carried out by recording 128 scans for each gradient step, with the in-house tdDOSYccbp.2D pulse sequence, which is based on the standard dstebpgp3s pulse sequence from Bruker (32). An in-house sequence uses bipolar gradients pairs to refocus chemical shift effects and compensate for chemical exchange that could lead to oscillations in the decay curve (33). The experiments were run using linear gradient of 32 steps between 2 and 95%, a diffusion time (Δ) of 0.2 s, and the length of the square diffusion encoding gradient pulses (δ) of 3 ms. The standard Bruker protocol was used for processing in TopSpin 3.2 software. The fitting of the diffusion dimension in the 2D-DOSY spectra was achieved using an exponential fit. In the processed 2D spectrum, the *y* axis shows the values of logD (*D* = diffusion coefficient), whereas the *x* axis shows the ^1^H chemical shift (in ppm scale).

### Electron Microscopy

For sample preparation, a freshly glow-discharged 300-mesh only–carbon coated grid (carbon film on copper 300 mesh; C300Cu100; EM Resolutions) is placed inside the chamber of a Vitrobot Mark II (FEI Company) at 8 °C for 30 s followed by fast plunging into liquid ethane. cryo-EM was performed on a JEM-2200FS/CR (JEOL Europe, Croissy-sur-Seine, France) transmission electron microscope equipped with a field emission gun operated at 200 kV and an in-column Ω energy filter. Digital images were recorded on a 4,000 × 4,000 (15 μm pixels) Ultrascan4000^TM^ charge-coupled device camera (Gatan Inc.) using DigitalMicrograph^TM^ (Gatan Inc.) software, at a nominal magnification of 60,000×, resulting in a final sampling of 1.7 Å/pixel.

### Mammalian cell culture and transfection

Human fibroblastoid M1 cell line (34) was grown in complete DMEM supplemented with 10% (v/v) fetal bovine serum, 0–1 mg/ml streptomycin, and 100 units/ml penicillin at 37 °C with 5% CO_2_ in incubator chamber. To maintain and amplify cells in a proper confluence, they were counted by means of an automated cell counter (CountessTM, Life Technologies) that performs cell count and viability calculations (from alive, dead, and total cells) using Trypan blue staining.

Transfection of a plasmid carrying the FAH-GFP constructs (pCMV6-AC-GFP) in M1 cells was performed using X-tremeGENE^TM^ HP DNA transfection reagent (Roche) following the manufacturer's instructions. The first day, 500,000 cells per P100 dish and plasmid construction were seeded in complete DMEM and allowed to attach for 24 h. On the second day, complete medium was replaced by 4 ml of Opti-MEM^TM^ reduced serum medium (Gibco, Ref. 31985). After 2 h, the medium was discarded, 1 ml of transfecting solution was poured drop by drop, and 3 ml of Opti-MEM^TM^ were added to completely cover the cells. After 4 h of incubation, 3 ml of complete DMEM was added. The following day, the medium was changed for complete DMEM supplemented with 2 mg/ml of G418, Geneticin^TM^ (Thermo Fisher, catalog no. 11811031) for clone selection.

### GFP fluorescence detection

The cells were seeded up to 50–80% confluence on glass coverslips for 24–48 h. Then the cells were fixed for 10 min with 2% (v/v) formaldehyde in PBS and washed twice with PBS. Finally, the coverslips were mounted onto glass slides on Fluoromont G (Southern Biotechnology Associates, Birmingham, AL) containing 0.7 g/ml 4′,6′-diamino-2-phenylindole to stain DNA (nucleus) to GFP direct detection. All procedures were carried out at room temperature. All immunostainings were analyzed in a fluorescent microscope (Zeiss Axiovert 200).

### FACS analysis

Flow cytometry analyses were performed using the BD FACSCanto TM II system (BD Biosciences). The data were analyzed using the BD FACSDiva^TM^ software (BD Biosciences) and later processed using Flowing software, a free flow cytometry data analysis software. All cells were harvested with TrypLE TM (Gibco) treatment; the cells were then washed twice with PBS and finally resuspended with D-PBS (400 μl) for flow cytometry analysis. GFP fluorescence was detected by FITC (maximum excitation, 494 nm; maximum emission, 520 nm) channel.

### Molecular dynamic simulations

With a sequence identity of 89% to *Homo sapiens*, the crystal structure of *Mus musculus* FAH was used for MD simulations (Protein Data Bank code 1QCN, chain A) (10). The atomic structure was then solvated in a rectangular water box of dimensions 104.8 × 104.5 × 95.46 Å^3^ using the Solvate plug-in of VMD (35). Na^+^ and Cl^−^ ions were added to the system to achieve charge neutralization up to a final concentration of 120 mm. The resulting system comprised ≈ 98,000 atoms. The CHARMM 36 force field was employed to describe the molecular mechanics (36). The TIP3P model was used for water. The standard CHARMM parameters were used for ions. NAMD version 2.10 was used for all calculations. Following 20,000 steps of energy minimization, the atomic system was equilibrated for 1 ns. Pressure was kept constant at 1 atm by the Nose–Hoover–Lagevin piston, with a damping time constant of 50 ps and a period of 100 ps. Temperature was maintained at 298 K by coupling the system to a Langevin thermostat, with a sampling coefficient of 2 ps^−1^. The multiple time step algorithm Verlet-I/r-RESPA was used to integrate the equations in motion. Nonbonded short-range forces were computed for each time step, whereas long-range electrostatic forces were updated every two time steps. G158D, I166G, and G158D mutants were introduced using PyMOL mutagenesis wizard. Simulation for WT, G158D, I166G, and G158D FAH was run for 100 ns. Atomic coordinates were collected every 20,000 steps. The MD trajectories were analyzed using VMD and in-house built scripts programmed in Matlab.

### Statistical analyses

The results are expressed as averages ± standard deviations.

## Author contributions

I. M., A. L., G. B.-S., D. G., and E. G. formal analysis; E. G. and J. M. F.-P. supervision; J. M. F.-P. and O. M. conceptualization; O. M. writing-original draft; O. M. writing-review and editing.

## Supplementary Material

Supporting Information

## Footnotes

3 The abbreviations used are:

HT1: hereditary tyrosinemia type I
FAH: fumarylacetoacetate hydrolase
FAA: fumarylacetoacetate
C-term: C-terminal domain
N-term: N-terminal domain
DOSY: diffusion ordered spectroscopy
GSTZ1: glutathione transferase ζ1
HGD: homogentisic acid dioxygenase
DMEM: Dulbecco's modified Eagle's medium.
