# Supplementary material for: Hereditary tyrosinemia type I–associated mutations in fumarylacetoacetate hydrolase reduce the enzyme stability and increase its aggregation rate
Source: J Biol Chem. 2019 Jul 12;294(35):13051–60. doi: 10.1074/jbc.RA119.009367 (PMC6721957; doi:10.1074/jbc.RA119.009367)
Supplement: Supporting Information [file supp_294_35_13051__index.html]

Hereditary tyrosinemia type I–associated mutations in fumarylacetoacetate hydrolase reduce the enzyme stability and increase its aggregation rate — The role of FAH stability in function and disease — Hereditary tyrosinemia type I–associated mutations in fumarylacetoacetate hydrolase reduce the enzyme stability and increase its aggregation rate — The role of FAH stability in function and disease — Supporting Information 

# Hereditary tyrosinemia type I–associated mutations in fumarylacetoacetate hydrolase reduce the enzyme stability and increase its aggregation rate

## Supporting Information

- Supporting Information (to be published online) - Supplementary material
