# Supplementary material for: Hereditary tyrosinemia type I–associated mutations in fumarylacetoacetate hydrolase reduce the enzyme stability and increase its aggregation rate
Source: J Biol Chem. 2019 Jul 12;294(35):13051–60. doi: 10.1074/jbc.RA119.009367 (PMC6721957; doi:10.1074/jbc.RA119.009367)
Supplement: Supporting Information [file supp_RA119.009367_152848_2_supp_361587_pch9lf.pdf]

Supplementary material for:

The role of fumarylacetoacetate hydrolase stability in hereditary tyrosinemia type I

Iratxe Macias[‡,1], Ana Laín[‡,1], Ganeko Bernardo-Seisdedos[1], David Gil[2], Esperanza Gonzalez[3], Juan M. Falcon-Perez[3,4], Oscar Millet[1,*]

[1]Protein Stability and Inherited Disease Laboratory, CIC bioGUNE, Bizkaia Technology Park, Bld. 800, 48160 Derio, Bizkaia, Spain.

[2]Electron Microscopy Platform, CIC bioGUNE, Bizkaia Technology Park, Bld. 800, 48160 Derio, Bizkaia, Spain.

[3]Exosomes Laboratory, CIC bioGUNE, Bizkaia Technology Park, Bld. 801A, 48160 Derio, Bizkaia, Spain.

[4]IKERBASQUE, Basque Foundation for Science, Bilbao, 48013 Spain.

Contents:

Table S1. Biophysical and biochemical properties of the pathogenic mutations under consideration.

Figure S1. Localization of the missense mutations in the structure of FAH.

Figure S2. Michaelis-Menten fit of the experimental enzymatic assay data.

Figure S3. Kinetic stability of FAH variants.

Figure S4. Circular dichroism data for P249T FAH and P342L FAH.

Figure S5. Structural rearrangements introduce by A134D, I166G and G337D observed by molecular dynamics.

Figure S6. Far-UV circular dichroism spectrum for WT FAH and all the mutations under consideration.

Table S2. Oligonucleotide primers used for the site directed mutagenesis.

FAH variant	Expression Yield / % of WT	Enzyme Activity / % of WT	ΔG_{F-U} / $kcal \cdot mol^{-1}$	$k_{Ag}(t)$ / h^{-1}	FAH Dimer / %
**WT**	100	100	9.5 ± 0.5	40 ± 2	94 ± 5
**M1V**	112 ± 5	103 ± 5	10.2 ± 0.5	38 ± 2	0
**N16I**	20 ± 2	n.d.	8.2 ± 0.4	28 ± 2	96 ± 6
**A35T**	10 ± 2	19 ± 1	9.1 ± 0.4	11 ± 1	92 ± 4
**F62C**	26 ± 4	23 ± 2	8.2 ± 0.3	17 ± 3	100 ± 5
**A134D**	111 ± 7	4 ± 1	8.4 ± 0.3	42 ± 3	89 ± 5
**G158D**	20 ± 3	0	7.1 ± 0.3	45 ± 4	68 ± 4
**V166G**	54 ± 5	68 ± 5	9.5 ± 0.4	33 ± 2	61 ± 4
**C193R**	81 ± 6	0.2 ± 2	6.6 ± 0.3	39 ± 2	86 ± 5
**G207D**	30 ± 4	13 ± 2	1.3 ± 0.1	41 ± 3	0
**D233V**	39 ± 5	2 ± 1	4.7 ± 0.4	19 ± 1	92 ± 5
**W234G**	21 ± 2	13 ± 3	7.1 ± 0.6	36 ± 2	75 ± 4
**P249T**	35 ± 4	15 ± 5	9.5 ± 0.5	39 ± 3	90 ± 5
**P261L**	16 ± 5	8 ± 3	6.9 ± 0.5	20 ± 2	94 ± 4
**T294P**	43 ± 5	45 ± 7	6.3 ± 0.6	40 ± 3	85 ± 5
**G337S**	83 ± 7	7 ± 2	5.7 ± 0.4	43 ± 4	28 ± 3
**P342L**	91 ± 6	98 ± 8	4.8 ± 0.4	38 ± 2	76 ± 5
**G343W**	31.2	11 ± 3	6.4 ± 0.5	17 ± 2	91 ± 5
**G369V**	35.1	8 ± 1	10.5 ± 0.6	14 ± 1	78 ± 4
**R381G**	45.0	12 ± 3	8.0 ± 0.5	40 ± 3	91 ± 5

Table S1. Biophysical and biochemical properties of the pathogenic mutations under consideration. For the expression yield, the enzyme activity and the unfolding free energy, the error bars represent the standard deviation from two independent duplicate experiments. For the aggregation constant, the error corresponds to the fitting error of the experimental data to a monoexponential decay curve. For the dimer composition, the error reflects the resolution of the diffusion coefficient determination from the DOSY experiment.

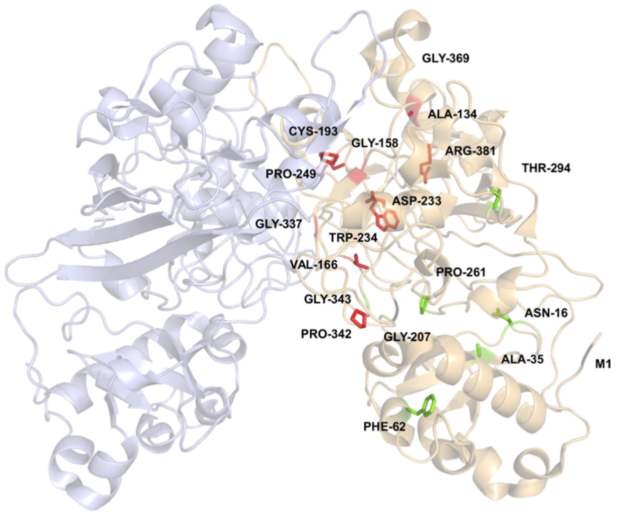

Figure S1. *Localization of the missense mutations in the structure of FAH.* The Gr1 set of missense mutations A134D, G158D, V166G, C193R, W234G, P249T, T294P, G337S, P342L, G369V and R381G are shown in red; the Gr2 group of mutants N16I, A35T, F62C, D233V, P261L and G343W are represented in green. M1V and G207D residue positions are colored in black.

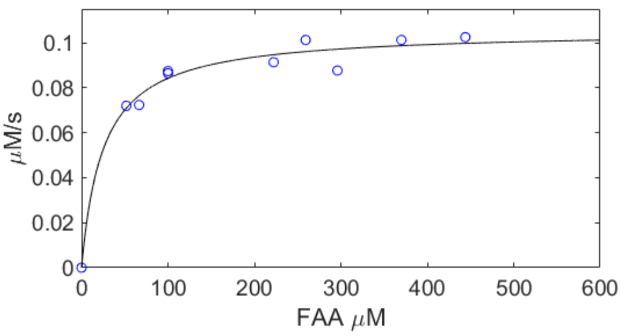

Figure S2. *Michaelis-Menten fit of the experimental enzymatic assay data.* Enzymatic conversion of FAA by the enzyme FAH (5 μM) at 37ºC (open blue circles). The solid line corresponds to the best fit to a Michaelis-Menten equation. The obtained parameters are: K_M = 25.2 ± 3 μM and k_{cat} = 0.10 ± 0.02 sec^{-1}.

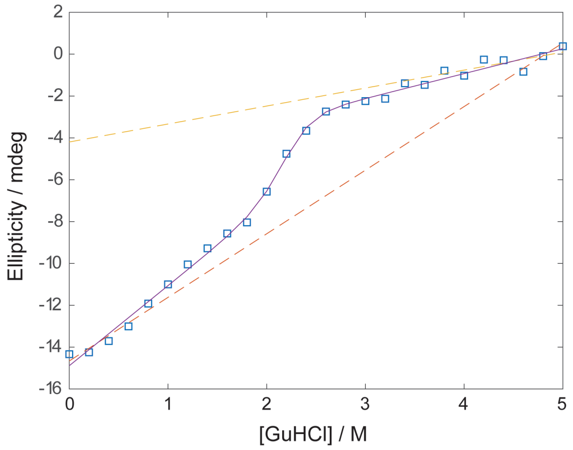

Figure S3. Guanidinium chloride (GuHCl) denaturation of WT FAH. Blue squares correspond to experimental data points, that are adjusted to the linear extrapolation model using the orange and yellow dashed lines as the denaturant linear dependence for the folded and the unfolded states respectively. The purple line shows the best fit to the experimental data using this model.

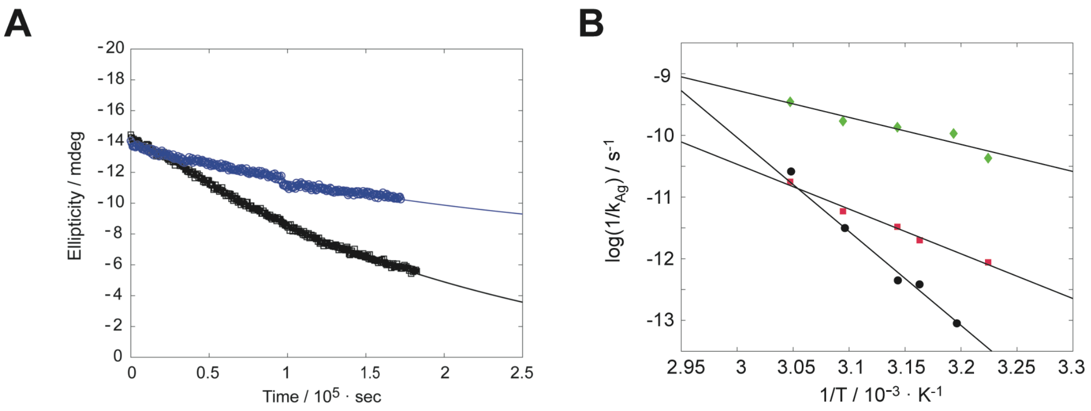

Figure S4. *Kinetic stability of FAH variants*. A) Variation in the ellipticity signal (proportional to secondary structure) over time for WT FAH (blue circles) and N16I FAH (black squares). The lines correspond to the best exponential fittings to the experimental data. B) Arrhenius plot for FAH aggregation constant (k_{Ag}) versus temperature (WT, black circles; G337S, green diamonds; P249T, red squares) at 10 μM. The solid line corresponds to the best fit of the magnitudes shown in the axes.

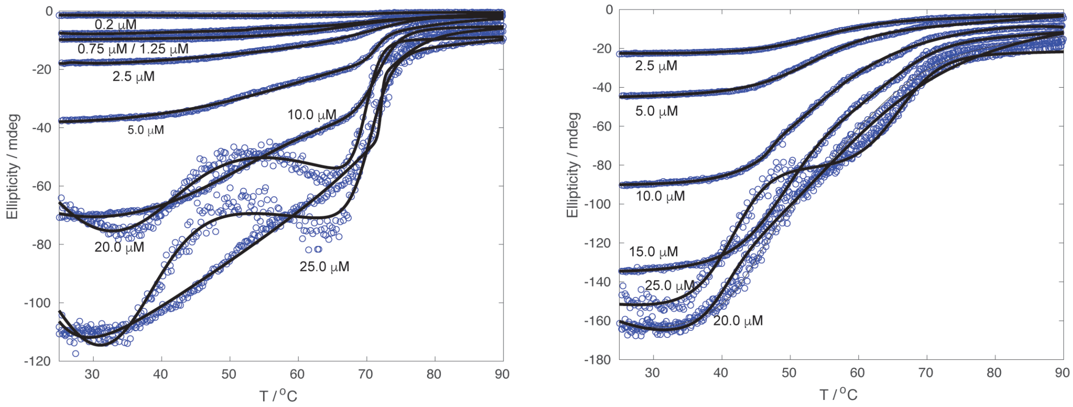

Figure S5. *Circular dichroism data for P249T FAH (left) and P342L FAH (right)*. Purple open circles correspond to the experimental data while solid black lines are the best collective fitting to the bi-modal thermal denaturation.

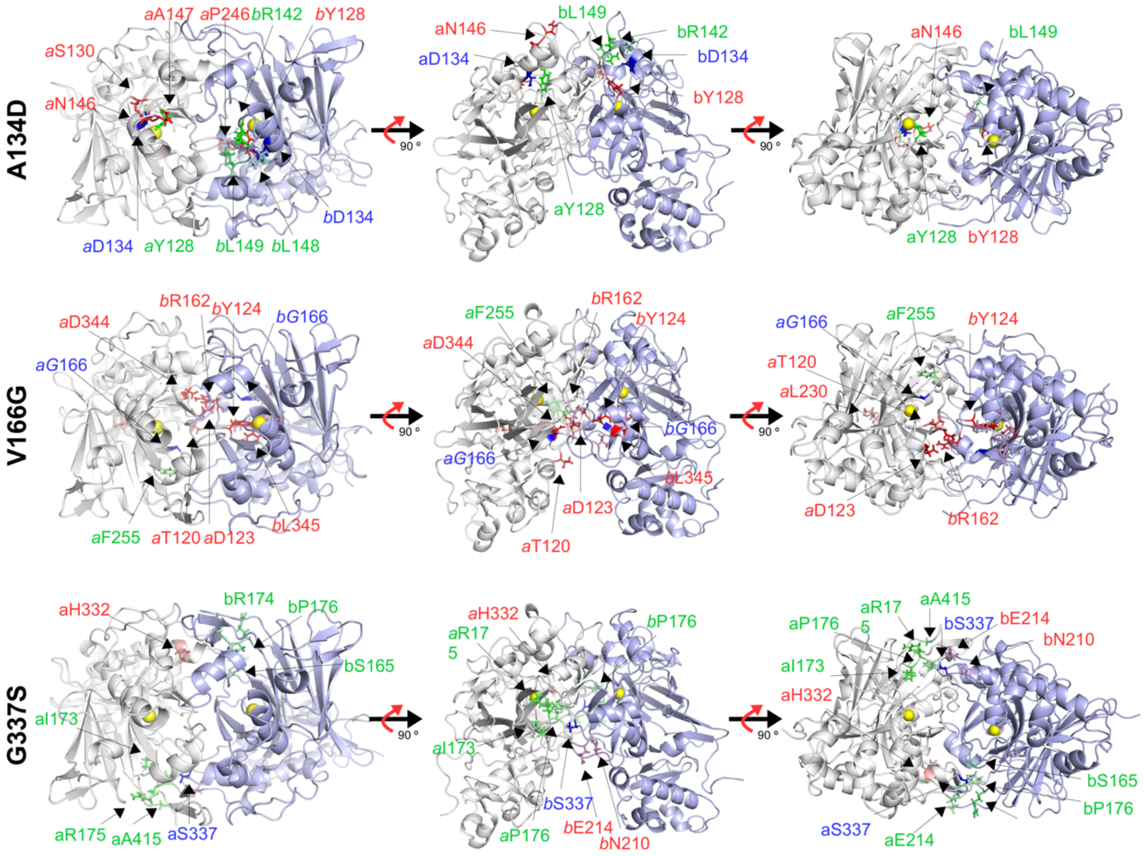

Figure S6. *Structural rearrangements introduce by A134D, I166G and G337D observed by molecular dynamics.* FAH chain *a* (grey) and chain *b* (light blue) are represented as cartoons. Mutated residue is highlighted as blue sticks. Affected neighboring residues are colored as green (gained contacts) or red (lost contacts) sticks. Color intensities will reflect the rate of newly created/lost contacts along the molecular dynamic simulation. Top (left panel), frontal (middle panel), and bottom views (right panel) are shown.

FAH variant	T_m [(1)]	ΔG_{F-U} [GuHCl]/ kcal·mol^{-1} [(2)]	ΔG_{F-U} [urea]/ kcal·mol^{-1} [(2)]	ΔG_{F-U} [T_m]/ kcal·mol^{-1} [(1)]
**WT**	68.0 ± 0.3	9.5 ± 0.5[(1)]	-	-
**M1V**	71.1 ± 0.7	-	-	10.2 ± 0.5
**N16I**	62.3 ± 0.5	8.2 ± 0.8	-	8.2 ± 0.4
**A35T**	66.0 ± 0.4	8.3 ± 0.8	9.9 ± 1.0	9.1 ± 0.4
**F62C**	62.0 ± 0.3	-	8.4 ± 0.8	8.2 ± 0.3
**A134D**	63.0 ± 0.4	7.4 ± 0.7	-	8.4 ± 0.3
**G158D**	57.2 ± 0.2	-	7.8 ± 0.8	7.1 ± 0.3
**V166G**	68.0 ± 0.4	7.1 ± 0.7	-	9.5 ± 0.4
**C193R**	55.4 ± 0.3	5.3 ± 0.5	5.8 ± 0.6	6.6 ± 0.3
**G207D**	34.3 ± 0.3	-	1.7 ± 0.2	1.3 ± 0.1
**D233V**	47.6 ± 0.3	-	4.5 ± 0.5	4.7 ± 0.4
**W234G**	57.2 ± 0.5	-	7.4 ± 0.7	7.1 ± 0.6
**P249T**	68.0 ± 0.4	-	9.9 ± 1.0	9.5 ± 0.5
**P261L**	56.4 ± 0.5	7.0 ± 0.7	-	6.9 ± 0.5
**T294P**	54.0 ± 0.5	-	7.4 ± 0.7	6.3 ± 0.6
**G337S**	51.7± 0.4	-	6.1 ± 0.6	5.7 ± 0.4
**P342L**	48.0 ± 0.3	5.1 ± 0.5	-	4.8 ± 0.4
**G343W**	54.5 ± 0.4	-	6.7 ± 0.6	6.4 ± 0.5
**G369V**	72.6 ± 0.5	-	10.0 ± 1.0	10.5 ± 0.6
**R381G**	61.3 ± 0.4	7.8 ± 0.8	-	8.0 ± 0.5

Table S2. Unfolding free energy determination using thermal or chemical denaturation, as indicated. For the expression yield, the enzyme activity and the unfolding free energy, the error bars represent the standard deviation from two independent duplicate experiments (1) or an estimation of the variation using duplicates for selected mutants ((2), error = 10%).

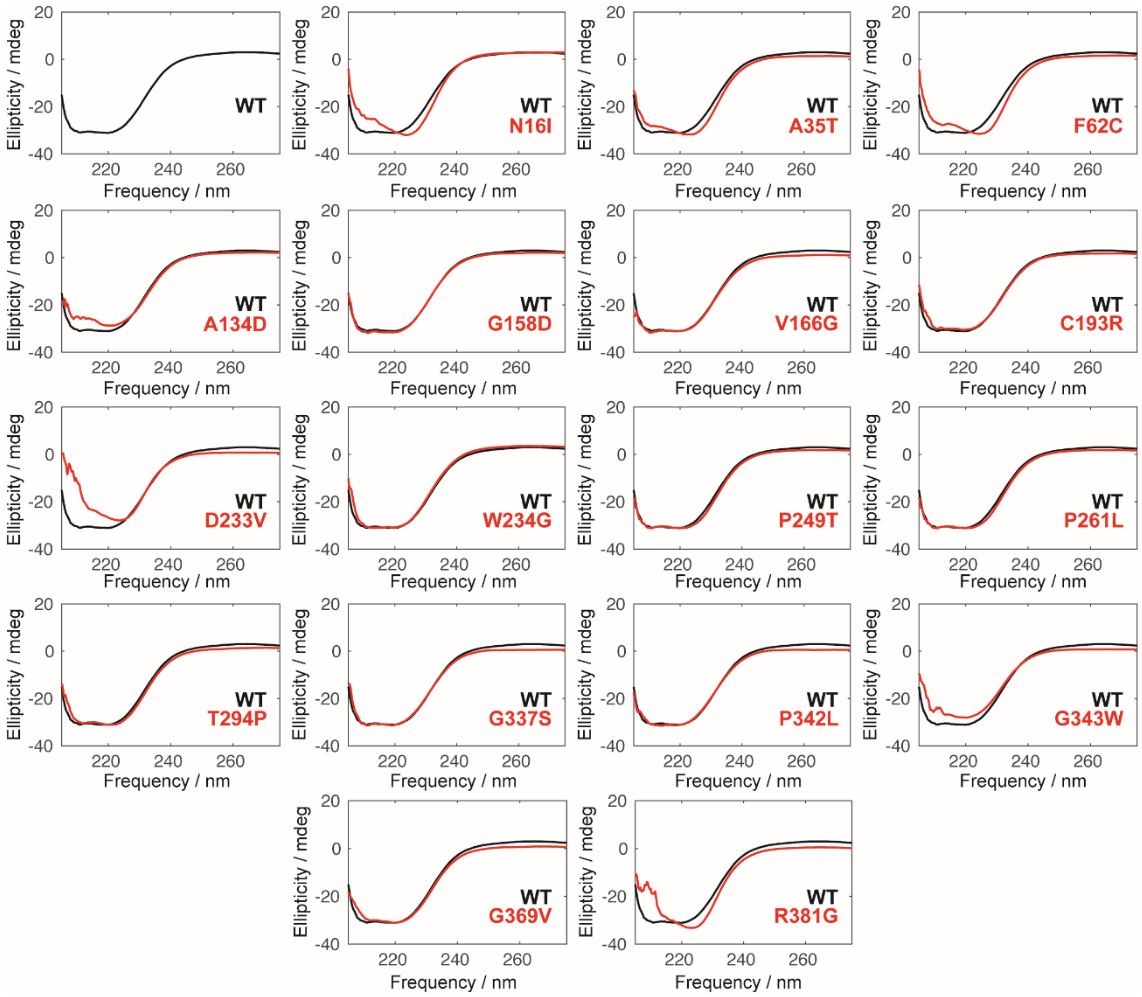

Figure S7. Far-UV circular dichroism spectrum for WT FAH (black) and all the mutations under consideration (red).

AA Change	Primer forward
M1V	GGT CGT CAT ATG GTGTCC TTC ATC CCG
N16I	TC CCT ATC CAC ATC CTG CCA TAC GG
A35T	CG CGT ATT GGT GTG ACC ATT GGC GAC CAG AT
F62C	AAA CAC CAG GAT GTC TGC AAT CAG CCT ACA CTC
A134D	TCT CGG CAG CAT GAT ACC AAC GTC GGA
G158D	AC TTA CCA GTG GAC TAC CAT GGC CG
V166G	CGT GCC TCC TCT GGC GTG GTG TCT GGC
C193R	GTA TAT GGT GCC CGC AAG CTC TTG GAC
G207D	GCT TTT TTT GTA GAC CCT GGA AAC CGT
D233V	TG GTC CTT ATG AAC GTC TGG AGT GCA CG
W234G	CTT ATG AAC GAC GGG AGT GCA CGT GAC
P249T	C CCT CTC GGG ACA TTC CTT GGG AAG AGT TTT G
P261L	ACT GTC TCT CTG TGG GTG GTG CCA ATG GAT GCT
T294P	GAC GAG CCT TAC CCA TTT GAC ATC AAC
G337S	CAC TCT GTC AAC AGC TGC AAC CTG CGG
P342L	C GGC TGC AAC CTG CGG CTG GGG GAC CTC CTG GCT TCT
G343W	TGC AAC CTG CGG CCG TGG GAC CTC CTG G
G369V	G TTG GAA CTG TCG TGG AAG GTA ACG AAG CCA ATT GAC C
R381G	GT CAG ACC GGG AAG TTT CTG CTG GAC GGG GAT G

Table S3. Oligonucleotide primers used for the site directed mutagenesis.
